# The Role of TLR2, TLR4, and TLR9 in the Pathogenesis of Atherosclerosis

**DOI:** 10.1155/2016/1532832

**Published:** 2016-10-04

**Authors:** Mohsin H. K. Roshan, Amos Tambo, Nikolai P. Pace

**Affiliations:** Department of Anatomy, Faculty of Medicine and Surgery, University of Malta, Msida, Malta

## Abstract

Toll-like receptors (TLRs) are key players in the pathogenesis of inflammatory conditions including coronary arterial disease (CAD). They are expressed by a variety of immune cells where they recognize pathogen-associated molecular patterns (PAMPs). TLRs recruit adaptor molecules, including myeloid differentiation primary response protein (MYD88) and TIRF-related adaptor protein (TRAM), to mediate activation of MAPKs and NF-kappa B pathways. They are associated with the development of CAD through various mechanisms. TLR4 is expressed in lipid-rich and atherosclerotic plaques. In TLR2^−/−^ and TLR4^−/−^ mice, atherosclerosis-associated inflammation was diminished. Moreover, TLR2 and TLR4 may induce expression of Wnt5a in advanced staged atheromatous plaque leading to activation of the inflammatory processes. TLR9 is activated by CpG motifs in nucleic acids and have been implicated in macrophage activation and the uptake of oxLDL from the circulation. Furthermore, TLR9 also stimulates interferon-*α* (INF-*α*) secretion and increases cytotoxic activity of CD4^+^ T-cells towards coronary artery tunica media smooth muscle cells. This review outlines the pathophysiological role of TLR2, TLR4, and TLR9 in atherosclerosis, focusing on evidence from animal models of the disease.

## 1. Introduction

The immune system is an integral component of body defense mechanisms. It is also a fundamental culprit in the pathogenesis of atherosclerosis, a chronic vascular disease that underlies the development of circulatory problems including coronary artery disease and cerebrovascular disease. In these scenarios, atherosclerosis runs a silent asymptomatic course, until stenosis or plaque rupture with superimposed thrombosis manifests clinically as stroke, myocardial infarction (MI), or death. Atherosclerosis involves a chronic and subclinical inflammatory process, initiated by endothelial damage and activation [1]. Subsequently, an elaborate cascade of steps involving various signaling molecules and leukocyte activation and migration follows, leading to the formation of atherosclerotic plaques. This complex process involves extensive interplay between various elements of the innate and adaptive immune systems.

The innate immune system constitutes the first line of defense against pathogens and is highly conserved and universal from an evolutionary perspective [2]. Central to the function of innate immunity is the recognition of pathogen-associated molecular patterns (PAMPs) or disease-associated molecular patterns (DAMPs) by immune-competent cells, such as tissue macrophages and endothelial cells. PAMPs are molecules of microbial origin, and DAMPs are endogenous molecules that normally are sequestered and become released in response to cell injury or endogenous stress signals, such as heat shock proteins (HSPs) [3]. PAMPs and DAMPs are recognized by a number of pattern recognition receptors (PRRs) expressed on sentinel immune cells, which can trigger inflammatory and adaptive immune reactions. Toll-like receptors (TLRs) constitute a major subgroup of pattern recognition receptors [4]. They were first identified in* Drosophila* mutants that exhibited susceptibility to fungal infections [5]. Subsequently, the human and murine homologs of* Toll* were identified, and studies showed their essential role in mediating the immune response against bacterial lipopolysaccharide (LPS) [6, 7].

Toll-like receptors are part of interleukin-1 receptor/toll-like receptor superfamily. They possess an extracellular N-terminal ligand-recognition domain with leucine-rich repeats and a cytoplasmic carboxyl terminal tail with toll/interleukin-1 receptor (TIR) signaling domains. The extracellular leucine-rich domain binds to PAMPs and forms homo- or heterodimers with other TLRs [8]. Vertebrate TLRs are grouped into six subfamilies based on sequence homology, and their structure has been extensively described elsewhere [9, 10]. At least thirteen mammalian TLRs are known, and these are expressed either on cell surfaces, where they bind microbial membrane components (TLR1, TLR2, TLR4, TLR5, and TLR6) or endosomally (TLR11, TLR12, and TLR13) where they recognize microbial nucleic acids (TLR3, TLR7, TLR8, and TLR9).

TLRs are intimately tied to the process of atheroma formation. TLR4 is expressed in human coronary plaques, and that baseline TLR4 macrophage expression is upregulated by oxidized LDL (oxLDL) [11]. TLR2 and TLR4 are expressed by macrophages, neutrophils, and dendritic cells and have been implicated in the development of coronary artery disease (CAD) through activation of NF-*κ*B pathways [12]. Furthermore, investigators have suggested that the phenomenon of molecular mimicry may be at play at activating innate immune pathways leading to atherosclerosis. TLR4 recognizes chlamydial antigens, and some studies have demonstrated a link between* Chlamydia pneumoniae* infection and atherothrombosis [13–15]. Although other investigators have failed to replicate this association [16, 17], it nevertheless provides an intriguing insight into the mechanistic link between TLR pathways and atheroma formation. This review article aims to describe the immunological role of TLRs in promoting the development of atherosclerosis, with a primary focus on the function of TLR2, TLR4, and TLR9.

*Pathophysiology of Atherosclerosis*. Central to the development and progression of atheromas is the interaction between activated monocytes and oxidized LDL (oxLDL) leading to formation of foam cells. Low density lipoprotein (LDL) represents two-thirds of total cholesterol [18]. The American College of Cardiology/American Heart Association define hyperlipidemia associated with increased cardiovascular disease risk as LDL plasma levels above 70–189 mg/dL (1.81–4.89 mmol/L) [19]. Moreover, an LDL/HDL ratio greater than 3 is considered atherogenic [18]. Plasma LDL is transported by apolipoprotein-B lipoprotein complexes in the circulation (ApoB-LPs) such as ApoB-100.* In vivo*, LDL-C undergoes oxidative modification resulting in the formation of oxLDL, which has strong proinflammatory and immunogenic properties. [20, 21]. Endothelial activation ensues following the lodging of oxLDL in the subendothelial matrix of the tunica intima [22]. Here, oxLDL triggers a response akin to that observed during chronic tissue injury, in that mononuclear leukocytes, primarily monocytes and lymphocytes, are recruited to the “site of injury” where oxLDL lodges [23, 24]. Tissue macrophages scavenge oxLDL and become transformed into foam cells. These lipid-laden foam cells form the basis of the initial lesion. The accumulation of foam cells gives rise to fatty streaks [25]. Furthermore, as the plaque evolves, intermediate lesions are formed, characterised by smooth muscle cell (SMC) proliferation and migration from the tunica media to the tunica intima. SMCs also become lipid-laden. Late stages of plaque evolution include atheromatous plaques and fibroatheromas [26]. In atheromas, foam cells undergo apoptosis and accumulate, forming a lipid-rich necrotic core [27]. Angiogenesis is evident in plaque development and its role is poorly understood. Some authors postulate that angiogenesis is triggered by cytokines secreted from activated macrophages to provide monocytes that can differentiate into tissue macrophages, to phagocytose the apoptotic foam cells [28]. Other authors propose that angiogenesis in these lesions serves to perfuse the hypoxic environment created by the cellular debris [29]. The consensus is that angiogenesis may set up a vicious cycle whereby there is a continual supply of monocytes which differentiate into tissue macrophages and become transformed into foam cells as a result of oxLDL uptake. On the other hand, fibroatheromas consist of extracellular matrix secreted by SMCs, and there is also evidence of calcified deposits. This is the basis of the fibrous cap formation [27]. In end-stage CAD, fibroatheromas may further evolve into complicated unstable lesions with surface defects and multiple necrotic cores. Complications from such lesions include erosion, rupture, fissure, and ulceration, resulting in various clinical manifestations, such as acute coronary syndromes [26, 30].

*Epidemiology of CAD*. CAD remains a prevalent cause of global mortality, accounting for an approximate 1.8 million annual deaths in Europe alone, which translates to 20% of total European mortality [31]. Low- and middle-income countries in Eastern Europe and Central Asia are now the most heavily affected by CAD. This is primarily due to the fact that these regions constitute the majority of the world's population. Contributing to the shift in CAD epidemiology is the increasing affluence in developing regions, including Latin America, Sub-Saharan Africa, Middle East, and South East Asia. The increasing urbanization, industrialization, and adoption of Western-style lifestyles that follows from economic growth impact negatively on cardiovascular mortality and have extensive socioeconomic implications [32]. Furthermore, the shift in population demographics resulting in increased life expectancy further compounds the clinical and public health burden exerted by CAD, particularly in Western countries [33].

## 2. TLR Signaling

TLRs are type 1 transmembrane glycoproteins comprised of extracellular, transmembrane, and intracellular signaling domains that are expressed either on the plasma membrane or on intracellular endolysosomal compartments. The cytoplasmic signaling domain is shared with that of the interleukin-1 (IL-1) receptor (the toll/IL-1R domain, TIR), and as a consequence of this homology TLRs activate pathways shared with IL-1R [12]. Upon ligand binding, the TLR signaling cascade is initiated by the TIR domain via a number of cytoplasmic adapter molecules. These include myeloid differentiation primary response protein (MyD88), TIR-domain-containing adaptor protein (TIRAP), and TRIF-related adaptor molecule (TRAM) [34].

The MyD88 pathway is essential for all TLR signaling, with exception of TLR3. MyD88 activates IL-1R associated kinases (IRAKs), IRAK-1 and IRAK-4, and TNF-receptor associated factor-6 (TRAF-6) [35, 36]. Consequently, recruitment of a number of proteins activates a complex containing TGF-*β*-activated kinase 1 (TAK1), TAK1-binding protein-1 (TAB1), TAB2, and TAB3 [37]. The TAK1/TAB complex in turn leads to activation of both the MAPK and NF-*κ*B signaling pathways [38]. These steps result in activation of a number of genes coding for proinflammatory cytokines and chemokines, including TNF-*α*, IL-1, and Il-6 [39].

TLR3 and TLR4 can also engage an MyD88-independent signaling pathway [40]. This requires TRIF and TRAM adapter proteins that lead to phosphorylation of interferon regulatory factor 3 (IRF3) and NF-*κ*B transcription factors [41]. The major outcome of TRIF-dependent TLR4 signaling is the production of type I interferons that have antiviral and antiproliferative activity [42, 43].

The balance between MyD88-dependent and TRIF-dependent TLR signaling is essential for proper immune function. These two pathways show reciprocal interaction and regulation. Wang et al. demonstrated that Nrdp1 (E3 ubiquitin ligase) inhibits the production of proinflammatory cytokines but increases IFN-*β* production in TLR-activated macrophages. This is achieved by suppressing the MyD88-dependent activation of NF-*κ*B through ubiquitination of MyD88 [44]. Conversely, Liu et al. showed that intracellular MHC class II molecules in antigen presenting cells can activate both MyD88 and TRIF cascades, leading to production of proinflammatory cytokines and interferons [45].

In addition to the activation of NF-*κ*B transcription factor in MyD88-dependent and MyD88-independent fashion, TLR2 has been implicated in the stimulation of proapoptotic pathways. Aliprantis et al. first demonstrated that bacterial lipoproteins induce monocyte apoptosis* in vitro* [46]. Subsequently they demonstrated that MyD88 is the common mediator of TLR2-induced apoptosis and NF-*κ*B activation and that TLR2 induces apoptosis through the FADD-caspase pathway in a manner analogous to members of the TNFR family [47, 48]. The* in vivo* relevance of TLR2 proapoptotic activity in either reinforcing or terminating the inflammatory response remains elusive, and its specific role in chronic inflammatory processes is poorly understood.

### 2.1. TLR Ligands

TLRs are able to bind a wide range of both endogenous and exogenous ligands. Primarily, they function as receptors for molecular domains borne on bacterial or viral pathogens. TLRs expressed on the plasma membrane recognize cell-wall components of bacteria and fungi, while those expressed on internal endolysosomal compartments bind viral PAMPs. Specifically, TLR2 binds bacterial lipoproteins, and TLR4 is primarily activated by bacterial lipopolysaccharide and TLR9 by unmethylated CpG nucleotide sequences [12]. In addition to exogenous ligands of microbial origin, TLRs are able to bind a wide range of endogenous ligands. These endogenous ligands are host-derived molecules that stimulate TLR signaling in the absence of infection, and they have been extensively reviewed elsewhere [49, 50]. Endogenous ligands include various extracellular matrix components, including fibronectin [51], fibrinogen [52], hyaluronic acid derivatives [53], heat shock proteins [54], and minimally oxidized low density lipoprotein [55]. These ligands are either actively released by cells at sites of injuy or passively released by cells damaged from inflamed tissues.

The evolving atherosclerotic plaque is a site of matrix turnover, tissue remodeling, and cell necrosis and hence contains a number of endogenous TLR ligands. Of particular interest are heat shock proteins (HSPs), which have been identified as powerful activators of innate immune function. Various investigators report that HSP60 induces a proinflammatory response in a TLR2 and TLR4 dependent fashion [56–58]. However, this hypothesis has been extensively challenged. Investigations showed that contamination by LPS in the HSP preparations is responsible for the observed TLR4 activation [59, 60]. LPS is a powerful inducer of TLR activation even in minute quantities, and LPS contamination results from the production of recombinant HSP in* Escherichia coli*.

**Figure 1:**
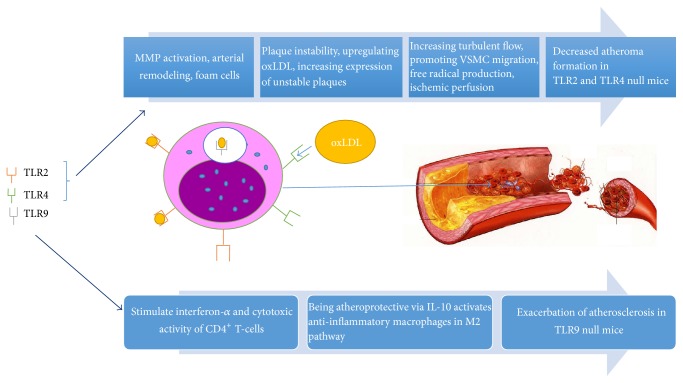
An overview of the proatherogenic effects of TLR2 and TLR4 compared to antiatherogenic effects of TLR9.

TLR signaling cascades can be activated by a broad range of host-derived molecules in the absence of exogenous infection, and this plays a central role in the development and progression of atherosclerosis. The subsequent sections of this review focus on the roles of TLR2, TLR4, and TLR9 in the pathogenesis of atherosclerosis.  Figure 1 outlines the key mechanisms from functional and animal studies implicating TLR2 and TLR4 in atherosclerosis.

### 2.2. TLR2 and Atherosclerosis

TLR2 is a cell surface receptor that binds a wide range of microbial components, such as gram-positive-derived lipoteichoic acid, bacterial lipoproteins, and zymosan [61]. It is expressed in a number of immune cells, the endothelium, and epithelial cells [62]. TLR2 has a unique ability to form functional heterodimers with either TLR1 or TLR6 resulting in relatively broad ligand specificity [63]. TLR2 expression, along with that of TLR1 and TLR4, is markedly increased in endothelial cells overlying atheromas [64]. Furthermore, endothelial TLR2 expression and activation may occur at areas of turbulent blood flow, such as the areas of lesion predilection within the aortic tree and heart.* In vitro* experiments using human coronary artery endothelial cells exposed to laminar blood flow showed lower levels of TLR2 expression when compared to endothelial cells exposed to static or turbulent flow [65]. Laminar flow at or above 0.5 N/m^2^ inhibits endothelial TLR2 expression via protein kinase CK2, which phosphorylates a transcription factor known as specificity protein-1 (SP1). This prevents SP1 binding to the TLR2 promoter, thereby reducing TLR2 expression. In doing so, flow suppression of TLR2 expression is considered to be atheroprotective [65]. Mullick et al. showed that, in atherosclerosis-susceptible LDLR-deficient (LDLR^−/−^) mice, complete deficiency of TLR2 leads to a reduction in atherosclerosis, whereas expression of TLR2 only on bone marrow derived cells has no impact on atherosclerosis [66]. This experiment showed that TLR2 expression on non-bone marrow derived cells, such as vascular endothelium, at sites of nonlaminar flow contributes to the atherosclerosis. Furthermore, the authors showed that on administration of Pam3CSK4 (a synthetic TLR2/TLR1 agonist), atherosclerotic burden was dramatically increased in LDLR^−/−^ mice. The proatherogenic effect of Pam3CSK4 was not observed in LDLR^−/−^ mice with complete TLR2 deficiency or in LDLR^−/−^ mice with a deficiency of TLR2 only in bone marrow derived cells. This finding reinforces the role of TLR2 in promoting atherosclerosis through its action in cells of non-bone marrow origin.

Further investigations by Mullick et al. using LDLR^−/−^ mice showed that aortic endothelial cell TLR2 expression was confined to areas of nonlaminar flow, specifically in the lesser curvature of the aorta, and that hyperlipidemia increases endothelial TLR2 expression [67]. Furthermore, the authors generated chimeric mice with green fluorescent protein (GFP) expression in BM derived cells (BMGFP^+^). Bone marrow reconstitution of* LDLR* ^−*/*−^ and* LDLR* ^−*/*−^ *TLR2* ^−*/*−^ mice with BMGFP^+^ cells showed that hyperlipidemia increases lesser curvature BMGFP^+^ leukocyte accumulation, lipid accumulation, and foam cell generation, whereas hyperlipidemic double mutant BMGFP^+^ *LDLR* ^−*/*−^ *TLR2* ^−*/*−^ mice had reduced lesser curvature atherogenic activity. This study showed that endothelial TLR2 expression is linked to early atherosclerosis in murine models.

The role of TLR2 in atherosclerosis has been reinforced by other studies. Using ApoE^−/−^ atherosclerotic mice Schoneveld et al. showed that exogenous TLR2 activation increases atherosclerotic plaque formation and plaque-media ratio. TLR2 is involved not only in the initial intimal lesion formation but also in development of occlusive disease [68]. Furthermore, TLR2 promotes vascular smooth muscle cell (VSMC) migration from tunica media to the intima in an IL-6 dependent manner [69]. Genetic deficiency of TLR2 reduces diet-induced atherosclerosis in ApoE^+/−^ mice [70], and TLR2 expression and activation regulates the inflammatory processes and ROS production following vascular injury in mouse models [71]. Aside from the involvement of TLR2 in atheroma development, evidence shows that TLR2 contributes to coronary endothelial dysfunction after ischemia/reperfusion by activating neutrophils and free radical production [72].

Scavenger receptors such as the CD36 coreceptor have been linked to activation of TLR2 [52]. CD36 functions in the recognition of various endogenous ligands, including oxLDL and the uptake of fatty acids. Nonetheless, even though TLR2 participates in the immune response to oxLDL, it is not a primary culprit in formation of foam cells unlike TLR4 and CD36 [73].

Other ligands implicated in TLR2 activation include high-mobility group box 1 protein (HMGB1). This is a nuclear transcription factor secreted by macrophages, monocytes, and dendritic cells that is expressed in atherosclerotic lesions [74]. It binds to TLR2 and triggers release of proinflammatory cytokines [75]. HMGB1 also has proatherogenic effects and stimulates macrophage migration in atherosclerotic lesions [55].

Members of Wnt family of glycoproteins such as Wnt5a have been reported to be coexpressed with TLR2 and TLR4 in macrophage rich regions of more advanced staged atheromatous plaques [76]. oxLDL can induce mRNA expression of Wnt5a, which correlates with the severity of atherosclerotic lesions in human studies [77]. Wnt5a has been implicated in the regulation of cholesterol transport in mouse macrophages [78]. Recently, Wnt5a pathways have shown playing a critical role in foam cell formation and oxLDL uptake [79]. The complex role of Wnt5a pathways in atherosclerosis has been extensively reviewed by Bhatt and Malgor [80]. Taken together, these studies highlight the elaborate signaling pathways involving TLR2 and its important role in driving atherogenesis.

### 2.3. TLR4 and Atherosclerosis

TLR4 resides in the plasma membrane, where it recognizes a number of exogenous ligands and activates a series of inflammatory cascades in an NK-*κ*B-dependent fashion [81]. Several lines of clinical and experimental evidence support its role in the pathogenesis of atheromas. Human studies showed that CD14^+^ monocyte TLR4 expression is increased in unstable angina and acute MI compared to control and stable angina groups [82, 83]. Satoh et al. showed that activation of TLR4 is associated with heart failure following MI [84]. Conversely, Tapp et al. showed that MI is associated with increased numbers of TLR4^+^ monocyte subsets, but not with higher TLR4 expression by individual monocytes [85].

Atherosclerotic plaque cells, in a manner analogous to TLR2, express TLR4 [64]. TLR4 levels are upregulated by oxLDL and Howell et al. showed that in murine models TLR4 is necessary for the oxLDL-induced macrophage differentiation into foam cells [86]. Similarly, TLR4 is a critical mediator in oxLDL-induced inflammatory cytokine expression in vascular smooth muscle cells [87]. Minimally modified LDL (a subtype of oxLDL that is essential for atherosclerosis) induces ROS production and macrophage cytoskeletal rearrangements in a TLR4 dependent and MyD88-independent manner [55, 88]. The spleen tyrosine kinase SYK binds to the cytoplasmic domain of TLR4 and mediates macrophage activation, membrane ruffling, macropinocytosis, lipid accumulation, and their consequent transformation into lipid-laden foam cells [89, 90].

Further experimental evidence from loss-of-function animal models supports the role of TLR4 in atherosclerosis. Atherosclerosis-prone ApoE^−/−^ mice with deficiency of TLR4 or MyD88 show attenuation in atherosclerosis development through decreased macrophage recruitment [91, 92]. Higashimori et al. showed that TLR4 contributes to early-stage intimal foam cell accumulation at lesion-prone aortic sites in ApoE^−/−^ TLR4^−/−^ mice, with a 75% reduction in intimal lipid levels compared to ApoE^−/−^ controls [93]. This study also showed that TLR4 is a more powerful contributor to foam cell formation than TLR2. Coenen et al. demonstrated that mice lacking macrophage TLR4 expression have reduced atherosclerotic lesion size when fed low-fat diets, despite no observed difference in body composition and plasma lipids [94]. TLR4 is also involved in outward arterial remodeling. This process compensates for loss of the vascular lumen due to plaque accumulation and involves collagen matrix degradation by matrix metalloprotease-9 (MMP-9), which is activated by TLR4 [95]. Disordered arterial remodeling contributes to atherosclerosis and vascular restenosis. TLR4^−/−^ mice show no outward arterial remodeling in carotid artery ligation and femoral artery cutoff models [96].

In addition to the established function of TLR4 in atheroma development, it also plays a critical role in the progression and eventual rupture of atherosclerotic plaques leading to the formation of occlusive thrombus. Ishikawa et al. demonstrated an increased expression of TLR4, but not TLR2, in ruptured human coronary atherosclerotic plaques, with TLR4 immunostaining observed in the infiltrating macrophages [97]. Recent literature further implicates TLR4 in plaque instability. Gargiulo et al. showed that specific components of oxLDL that accumulate in atheromas enhance the release of proinflammatory cytokines and upregulate MMP-9 in a TLR4/NF-*κ*B-dependent fashion [98]. This investigation directly implicates the role of lipid derivatives as endogenous TLR4 ligands that contribute to matrix breakdown.

The extracellular matrix (ECM) is a key player in the progression of atherosclerotic disease, and several studies have described the structural alterations that develop during atherosclerosis [83, 99]. Chronically inflamed tissues express extracellular matrix (ECM) proteins that regulate the migration of leukocytes and other immune cells to sites of injury. While healthy human endothelium rests on an ECM composed of collagen IV and laminin, the ECM of atherosclerotic vessels contains abundant fibronectin in both human and mouse models [100, 101]. Fibronectin occurs in two forms generated by alternative splicing, plasma fibronectin (pFN) and cellular fibronectin (cFN). pFN is a soluble dimer secreted by hepatocytes. cFN is expressed in the ECM of various tissues and is a multimer that forms fibrils and contains extra domains A and B (EDA and EDB) [102]. cFN is synthesised by vascular smooth muscle and endothelial cells. The extra domain A in fibronectin (EDA^+^FN) is implicated in a number of biological processes, including atherosclerosis. Deletion of the alternatively spliced EDA exon reduces the number and size of atherosclerotic lesions in ApoE null mice [103, 104].* In vitro* studies also suggested that the FN EDA domain activates TLR4 [51]. Further investigations using ApoE null mice with either constitutive expression or knockout of the fibronectin EDA domain showed that EDA^+^FN promotes progression of atherosclerosis through a mechanism that is partially dependent on TLR4 [105]. The authors showed that EDA^+^FN drives macrophage recruitment into developing plaques through TLR4 signaling. Comparably, Prakash et al. showed that platelet TLR4 facilitates the prothrombotic effects of cellular EDA^+^FN on platelet aggregation and arterial thrombosis [106]. EDA^+^FN mice lacking platelet TLR4 showed decreased thrombus formation and a slower thrombus growth rate compared with control mice expressing platelet TLR4, further highlighting the proatherogenic effect of the FN-TLR4 interaction.

### 2.4. TLR9 and Atherosclerosis

While TLR2 and TLR4 are expressed on the cell surface, TLR9 colocalises to the endoplasmic reticulum in various cell types, including B-cells, macrophages, dendritic cells, and plasma cells [107]. In the ER, TLR9 is able to detect and bind unmethylated oligodeoxynucleotide CpG motifs in microbial DNA sequences and trigger inflammatory responses [108]. Unmethylated CpG sequences are rare in eukaryotic genomes but abundant in prokaryotes.

Apart from its role in the recognition of bacterial DNA, TLR9 has been closely linked with the development of atherosclerotic lesions, since it is activated by CpG motifs in nucleic acids that are released during vascular necrosis. Activation of TLR9 stimulates the transformation of murine macrophages into foam cells in an NF-*κ*B- and IRF7-dependent manner [109]. This process is inhibited by the activation of liver x-receptors, which are transcriptional regulators of lipid and carbohydrate metabolism [110].

In addition, activation of TLR9 via CpG-containing nucleotide sequences in plasmacytoid dendritic cells stimulates interferon-*α* (INF-*α*) secretion and increases the cytotoxic activity of CD4^+^ T-cells towards vascular smooth muscle cells [111].

However, some investigators have suggested that TLR9 is protective against atherosclerosis.* In vitro* activation of TLR9 stimulates interleukin-10 (IL-10) production, which in turn inhibits the expression of INF-*α* secreted by plasma dendritic cells and inhibits CD4^+^ CD25^+^ T-cell proliferation [112, 113]. Loss-of-function animal models have been used to further elucidate the role of TLR9 in atheroma. Koulis et al. used a double knockout mouse model lacking both TLR9 and ApoE to compare aortic sinus atherosclerotic lesion development. The investigators showed a 33% increase in lipid deposition and atherosclerotic plaque size in ApoE^−/−^/TLR9^−/−^ mice compared to ApoE^−/−^ mice [114]. Furthermore, there was significant accumulation of macrophages, dendritic cells, and INF-*α* in vasculature of ApoE^−/−^/TLR9^−/−^ mice compared to control animals. Loss of TLR9 function thus exacerbates atherosclerosis in ApoE null mice exposed to a high fat diet.

Other investigators have reported contradictory findings. Pharmacologic inactivation of TLR9 pathways in animal models results in a reduction in atherosclerotic lesion generation in ApoE^−/−^ mice and reduced instability of vulnerable plaques [115]. Ma et al. showed that inactivation of TLR9 using immunoregulatory oligodeoxynucleotides such as IRS869 reduces plaque burden and shunts the activities of proinflammatory macrophages (M1) into anti-inflammatory macrophages (M2). Krogmann et al. investigated the effect of administering intravenous ODN1826 (type B oligodeoxynucleotide that activates TLR9). They showed that stimulation of TLR9 impairs reendothelialization following acute vascular injury and increases plaque development in ApoE^−/−^ mice [116]. Nonetheless, pharmacologic TLR9 activation did not alter the endothelium-dependent vasodilation, suggesting that TLR9 activation only affects the regenerative process but not the vasoactive function of endothelial cells. Both pro- and antiatherosclerotic effects of TLR9 have been described. One possible explanation for the discrepancies between the investigations reported by Krogmann et al. includes differences in the oligodeoxynucleotide dosage regime, as development of atherosclerosis requires chronic sustained inflammatory trigger [116]. Further investigations are needed to further define the role of TLR9 agonists in atherosclerotic disease.

### 2.5. Regulatory T-Cells and TLR Signaling in Atherosclerosis

Atherosclerosis is a highly complex process that involves multiple signaling cascades and a wide variety of cell types, including monocytes, macrophages, VSMCs, and various subtypes of T lymphocytes. T-cell subtypes exert differing effects on atherogenesis. Th1 cells are proatherogenic while Th2 and Tregs (T-regulatory cells) have atheroprotective effects [117, 118]. The function of different T-cell subtypes in atherosclerosis has been recently reviewed elsewhere [119, 120]. Of particular interest is the link between Tregs and atherosclerosis. Subramanian et al. investigated the role of monocyte-derived CD11c^+^ dendritic cells. In this study, LDLR^−/−^ mice were reconstituted with bone marrow cells where CD11c^+^ cells lacked the TLR adaptor MyD88 [121]. This decreases their ability to activate T-effector cells and was expected to produce a decrease in atherosclerosis [122]. On the contrary, the study demonstrated an increase in aortic root atheroma size and monocyte infiltration, with no differences in lipoprotein levels. This effect was due to the loss of Treg-mediated suppression of MCP-1, and the data demonstrated the atheroprotective role of Treg cells in murine models. It also provides interesting insight into the function of TLRs at the crossroads of innate and adaptive immunity in atherosclerosis, which might be useful in the development of novel therapeutic strategies [123].

### 2.6. TLR Polymorphism in Atherosclerosis

Further evidence supporting the role of TLRs in atheroma development comes from genetic association studies. Most studies have focused on two missense polymorphism types in TLR4, Asp299Gly and Thr399Ile, and conflicting associations have been reported. Some investigators reported a reduced risk of atherosclerosis in carriers of the Asp299Gly TLR4 polymorphism [124], which was not reproduced in other investigations [125]. Other case-control candidate genes have identified an association between the TLR4 polymorphism and acute myocardial infarction, with a reduced incidence of cardiovascular events in the 299GLy allele [126, 127]. However, a meta-analysis by Koch et al. showed no association between TLR4 variants and myocardial infarction in a Caucasian cohort [128]. Population-specific differences in risk allele frequency possibly account for the different associations reported in the literature [129, 130].

## 3. Conclusion

TLRs form part of the innate arm of the immune system where they play an integral role in defense mechanisms against pathogens. They are expressed on a number of immune cells including macrophages, monocytes, and dendritic cells. TLRs distinguish between host molecules and PAMPs upon binding to the ligand and thereby trigger inflammatory response via NK-*κ*B pathways. MyD88 plays a critical role in TLR signaling. MyD88 deficiency in myeloid cells has been shown to inhibit macrophage recruitment and activation of the M1 system.* In vivo* and* ex vivo* studies have linked MyD88-dependent growth factors produced by endothelial cells to initiating inflammation and development of atherosclerosis by priming the monocytes in arterial and adipose tissues to differentiate into M1-proinflammatory macrophages instead of M2 anti-inflammatory macrophages.

Different subtypes of TLRs are involved in different aspects of inflammatory response. TLR2 and TL4 are strongly implicated in atheroma development and progression, leading to coronary artery disease. This is evidenced by a number of* in vitro* and animal studies using atherosclerosis-prone LDLR or ApoE deficient mice. Moreover, the development of atherosclerotic plaques often requires more than just TLRs, and coreceptors such as CD36 have been linked to TLR2 activation and inducing atherosclerotic change. Even though TLR2 and TLR4 are often labelled as “atherogenic promoters,” they are not considered as the primary culprit behind certain inflammatory changes such as formation of foam cells and generation of oxLDL. Furthermore, the presence of TLR4 in unstable plaques suggests that it is a major driver of CAD progression. TLR4 upregulates matrix metallopeptidases such as MMP-9 which makes plaques prone to rupture. The precise function of TLR9 in atherosclerosis is yet to be defined, with conflicting studies reporting both proatherogenic and antiatherogenic effects. Clearly, TLRs are promising therapeutic targets and possible biomarker candidates for a wide range of pathologies, but further studies are required in order to fully define their function.

## Abbreviations

CAD: Coronary arterial disease
NK-*κ*B: Nuclear factor kappa-light-chain enhancer B-cells
TLRs: Toll-like receptors
CD4^+^ T-cells: Cluster of differentiation 4 T-cells
ApoE^−/−^: Absence of apolipoprotein E
Wtn5a: Wingless-type MMTV integration site family member 5a
MyD88: Myeloid differentiation primary response gene
PRRs: Pattern recognition receptors
OxLDL: Oxidized low density lipoprotein
MM-LDL: Minimally modified low density lipoprotein
PAMPs: Pathogen-associated molecular patterns
DAMPs: Disease-associated molecular patterns
VSMCs: Vascular smooth muscle cells
CVD: Cardiovascular disease
TIRAP: TIR-domain-containing adaptor protein
TRAM: TIRF-related adaptor molecule
SP1: Specificity protein-1
NBM: Non-bone marrow origin
TNF: Tumour necrosis factor
IL-1/IL-6/IL-18: Interleukin-1/interleukin-6/interleukin-18
MMP-9: Metalloproteinase-9
ECs: Endothelial cells
ECM: Extracellular matrix
FN: Fibronectin
EDA: Extra domain A of fibronectin
ODN: Oligodeoxynucleotide
TNF-*α*: Tumour necrosis factor-alpha
BM: Bone marrow
LPS: Lipopolysaccharides
HDL: High density lipoprotein
Pam3/CSK4 ETC: N-Plamitoyl-(S)-[2,3-bis(palmitoyloxy)-(2RS)-propyl]/Csy-Ser-Lys4 (Pam3/CSK4).
